# Metabolomics approach reveals metabolic disorders and potential biomarkers associated with the developmental toxicity of tetrabromobisphenol A and tetrachlorobisphenol A

**DOI:** 10.1038/srep35257

**Published:** 2016-10-13

**Authors:** Guozhu Ye, Yajie Chen, Hong-ou Wang, Ting Ye, Yi Lin, Qiansheng Huang, Yulang Chi, Sijun Dong

**Affiliations:** 1 Key Laboratory of Urban Environment and Health, Institute of Urban Environment, Chinese Academy of Sciences, Xiamen 361021, China; 2 University of Chinese Academy of Sciences, Beijing 100049, China

## Abstract

Tetrabromobisphenol A and tetrachlorobisphenol A are halogenated bisphenol A (H-BPA), and has raised concerns about their adverse effects on the development of fetuses and infants, however, the molecular mechanisms are unclear, and related metabolomics studies are limited. Accordingly, a metabolomics study based on gas chromatography-mass spectrometry was employed to elucidate the molecular developmental toxicology of H-BPA using the marine medaka (Oryzias melastigmas) embryo model. Here, we revealed decreased synthesis of nucleosides, amino acids and lipids, and disruptions in the TCA (tricarboxylic acid) cycle, glycolysis and lipid metabolism, thus inhibiting the developmental processes of embryos exposed to H-BPA. Unexpectedly, we observed enhanced neural activity accompanied by lactate accumulation and accelerated heart rates due to an increase in dopamine pathway and a decrease in inhibitory neurotransmitters following H-BPA exposure. Notably, disorders of the neural system, and disruptions in glycolysis, the TCA cycle, nucleoside metabolism, lipid metabolism, glutamate and aspartate metabolism induced by H-BPA exposure were heritable. Furthermore, lactate and dopa were identified as potential biomarkers of the developmental toxicity of H-BPA and related genetic effects. This study has demonstrated that the metabolomics approach is a useful tool for obtaining comprehensive and novel insights into the molecular developmental toxicity of environmental pollutants.

Tetrabromobisphenol A (TBBPA) and tetrachlorobisphenol A (TCBPA) are halogenated derivatives of bisphenol A (H-BPA); and are used as flame retardants worldwide^1^,^2^. Owing to their high levels of production, widespread usage, low volatility, high lipophilicity and recalcitrance, TBBPA and TCBPA persist in the environment and have been detected in wildlife, human serum, umbilical blood and breast milk^3^,^4^,^5^.

Major sources of human exposure to TBBPA and TCBPA mainly include dust ingestion, diet, dermal contact and air inhalation^6^. TBBPA exposure levels in infants were found to be 2, 3, 5 and 13 times higher than those in toddlers, children, teenagers and adults, respectively^6^,^7^. Additionally, the average level of TBBPA intake via human milk in nursing infants aged 1–6 months was 19.9 times higher than that in adults^8^. Moreover, the average mother-to-infant TBBPA transfer ratio was 3.04, and TBBPA levels were 2–5 times higher in infants aged 1–3 months than in mothers, and decreased significantly with age^9^. Notably, fetuses and infants were more vulnerable to environmental toxins than the other age groups^10^. Accordingly, the potential toxic effects of TBBPA and TCBPA on the development of fetuses and infants are worthy of comprehensive investigations.

Accumulating data have demonstrated the toxic effects of H-BPA on biological development, in addition to the reproductive, nervous and endocrine system^2^,^11^,^12^,^13^,^14^,^15^. As illustrated in zebrafish, TBBPA exposure cause trunk edema, tail malformations, delayed hatching time, decreased hatching rates, and increased mortality of embryos and larvae^12^,^15^. A one-generation reproduction study of Wistar rats revealed delayed sexual development in females due to TBBPA exposure^16^. Moreover, increased DNA damage and apoptosis of testicular cells have been reported in mice exposed to TBBPA^14^. However, the molecular mechanisms underlying the developmental toxicity of H-BPA are unclear. To the best of our knowledge, metabolomics studies examining the developmental toxicity of H-BPA have not been conducted.

The marine medaka (Oryzias melastigma) model has many advantages, such as a short generation time, transparent eggs that facilitate experimental observations and manipulations, high levels of egg production, eggs and larvae that are sensitive to environmental pollutants, and the fact that it has been widely applied in toxicology studies^17^. Accordingly, O. melastigma embryos were used as a model to elucidate the potential effects of H-BPA on developmental toxicity in a comprehensive manner by employing metabolomics based on gas chromatography-mass spectrometry (GC-MS), which has been shown to be a useful approach for discovering metabolic disorders related to environmental toxicology^18^,^19^. First, embryos were exposed to TBBPA and TCBPA to evaluate the developmental toxicity of H-BPA. Subsequently, F1 embryos were collected for a metabolomics analysis to determine the developmental toxicology of H-BPA and related genetic effects after F0 and F1 exposure to H-BPA. The aim of the present study was to provide the first comprehensive and novel understanding of metabolic disorders and potential biomarkers associated with the developmental toxicity of H-BPA and related genetic effects.

## Results

### Developmental toxicity of H-BPA in embryos

After embryonic exposure to TBBPA and TCBPA at 50, 200 and 800 μg/L, respectively, morphological lesions, such as pericardial and abdominal edemas, developed in O. melastigma embryos and larvae (n = 3, Supplementary Fig. S1a–c). The hatchability of the embryos at 7 dpf also significantly decreased in response to exposure to 50, 200, and 800 μg/L of TCBPA and 800 μg/L of TBBPA (n = 3, *p* < 0.05, Supplementary Fig. S1d). Moreover, the hatchability was significantly reduced after exposure to 800 μg/L of TCBPA (n = 3, *p* < 0.05, Supplementary Fig. S1d). These data clearly demonstrate that embryonic exposure to H-BPA can induce developmental lesions and retardation, as evidenced by the response of the zebrafish to TBBPA exposure, and that developmental toxicity of TCBPA is more potent than TBBPA^12^. Owing to the presence of significant developmental retardation at 7 dpf, F1 embryos at 6 dpf were subsequently collected for a metabolomics analysis to examine the developmental toxicology and related genetic effects of H-BPA on F0 and F1, respectively (Supplementary Fig. S1e).

### Analytical performance of metabolic profiling

Several criterions for evaluating analytical performance of metabolic profiling suggest that QC samples should be tightly clustered, and that 60% of the metabolites in the metabolomics data should be located within 15% of the mean, with a more lenient criterion (within 20%) for metabolites at or near their limits of quantification^20^,^21^,^22^. In our study, the 4 QC samples clustered closely in PCA score plot of the samples, and RSDs (relative standard deviations) of 90.7, 93.8 and 96.4% of the 2860 ion peaks were less than 15, 20 and 30% in the QC samples, respectively (Supplementary Fig. S2). Accordingly, metabolic profiling in the present study was highly reproducible and stable.

### Global metabolic disorders in response to H-BPA exposure

The PLS-DA results showed that metabolic profiling of the H-BPA exposure groups differed greatly from that of the control group; and that clear differences were present between the 50 and 200 μg/L treatment groups (Supplementary Fig. S3). However, the low dose (50 μg/L) is closer to the controls than the high dose (200 μg/L) in every group, which might indicates that most of the metabolic changes from 0, 50 to 200 μg/L H-BPA treatment were not dependent on concentration. Subsequently, 1605 differential ions were discovered based on comparisons between the control and treatment groups using the Mann-Whitney U test to identify metabolic disorders associated with the developmental toxicology of H-BPA (*p* < 0.05). In total, 82 differential metabolites were identified, and 75 metabolites were further verified according to the reference standards (*p* < 0.05, Supplementary Table S1). The heat map plot showed that most of the differential metabolites were significantly decreased in embryos after exposure to TBBPA and TCBPA (*p* < 0.05), and that metabolic disorders induced by TBBPA were similar to those induced by TCBPA. Furthermore, metabolic disorders in offspring embryos resulting from parental TBBPA exposure were also similar to those in offspring embryos owing to parental TCBPA exposure (Supplementary Fig. S4). Collectively, H-BPA exposure could induce large metabolic disturbances; and the effects of TBBPA exposure were similar to those of TCBPA exposure.

Subsequently, pathway analysis was used to discover the metabolic pathways affected by H-BPA exposure. Significant disturbances in certain metabolic pathways were found in embryos exposed to TBBPA and TCBPA, including pathways related to amino acid metabolism (i.e., alanine, aspartate and glutamate metabolism, phenylalanine, tyrosine and tryptophan metabolism, valine, leucine and isoleucine metabolism, arginine and proline metabolism, among others), glycolysis, the tricarboxylic acid (TCA) cycle, purine metabolism, and lipid metabolism (Supplementary Fig. S5). Furthermore, parental exposure to TBBPA and TCBPA also led to disturbances in amino acid metabolism, the TCA cycle, glycolysis, purine metabolism, lipid metabolism and other pathways in offspring embryos, demonstrating the heritability of metabolic disorders resulting from H-BPA exposure (Supplementary Fig. S6). Detailed major changes in metabolic pathways are discussed below.

### Disorders of glycolysis in response to H-BPA exposure

**Figure 1:**
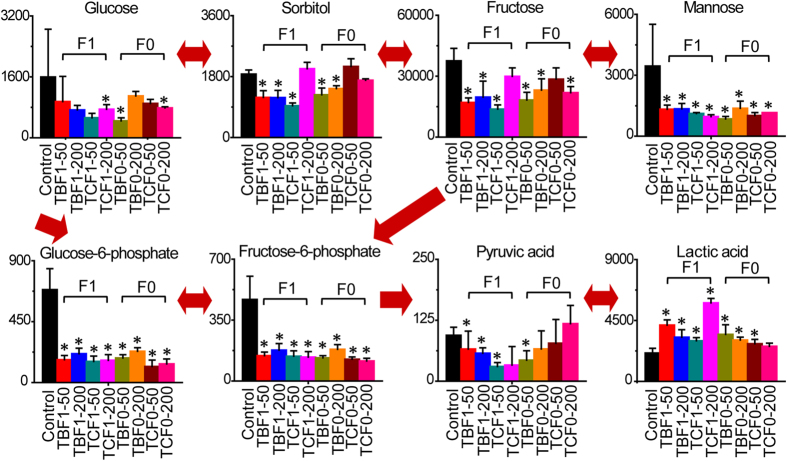
Disorders of glycolysis in response to H-BPA exposure. Metabolite levels are represented as the mean + sd. *p < 0.05, n = 4, two-tailed Mann-Whitney U test.

After exposure to H-BPA, significant disorders in glycolysis were clearly evident via pathway mapping with the differential metabolites (Fig. 1). Mannose, fructose, glucose-6-phosphate, fructose-6-phosphate and pyruvate were all significantly decreased, whereas lactate was significantly increased in response to TBBPA and TCBPA exposure. Furthermore, mannose, fructose, glucose-6-phosphate, fructose-6-phosphate and lactate were also significantly altered in the offspring embryos following parental exposure to H-BPA. These data demonstrated that glycolysis was significantly up-regulated in embryos in response to H-BPA exposure; and that the effect of H-BPA on glycolysis was heritable. The enhanced glycolysis indicated the presence of an acidic environment and energy disturbances in embryos in response to H-BPA exposure. Moreover, a significant increase in lactate accumulation suggested the presence of neural activation in H-BPA-exposed embryos^23^,^24^,^25^.

### Disorders of TCA cycle in response to H-BPA exposure

**Figure 2:**
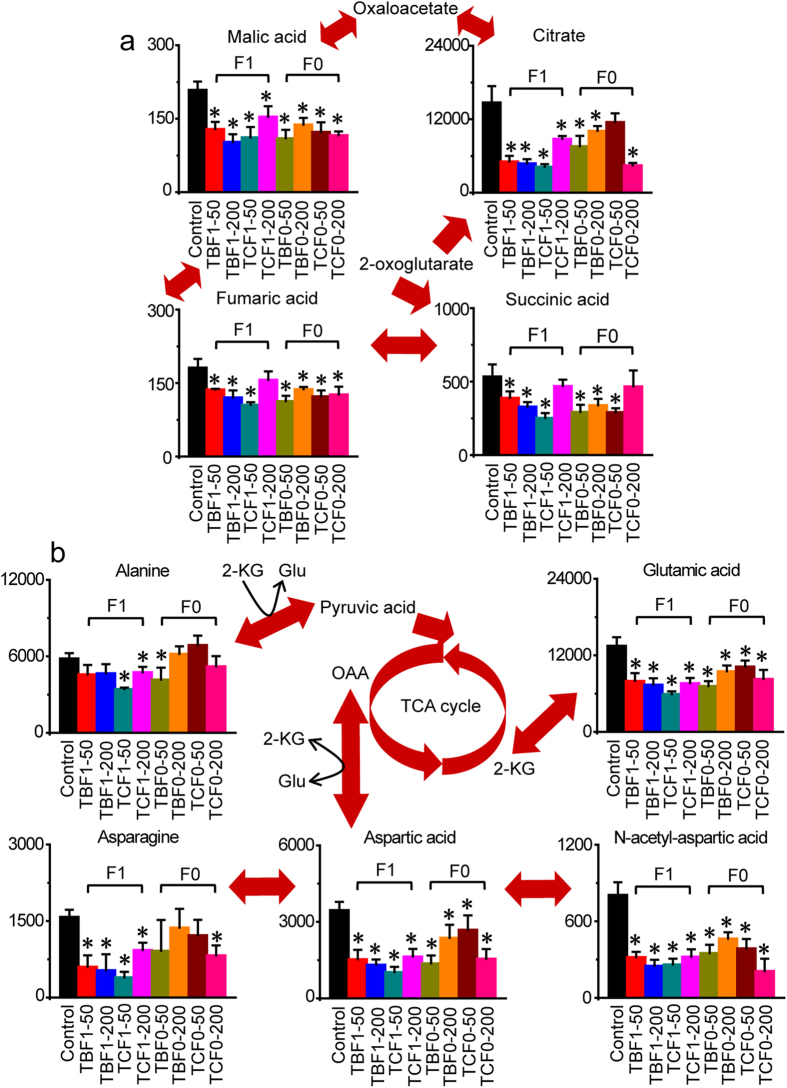
Disorders of the TCA cycle, and alanine, aspartate and glutamate metabolism in response to H-BPA exposure. Metabolite levels are represented as the mean + sd. *p < 0.05, n = 4, two-tailed Mann-Whitney U test.

Significant disturbances were evident in the TCA cycle owing to H-BPA exposure (Fig. 2a). Citrate, fumarate, malate, and succinate were all significantly decreased in embryos after exposure to TBBPA and TCBPA. Decreases in malate, citrate, fumarate and succinate were also observed in offspring embryos in response to parental exposure to TBBPA and TCBPA. The data revealed that the TCA cycle was inhibited, and that the inhibitory effects of H-BPA exposure were heritable. The decreases in TCA cycle activity revealed a reduction of the carbon and nitrogen sources for the production of energy and biosynthetic precursors of amino acids, lipids and pyrimidines in response to H-BPA exposure. Abnormal mitochondrial function is unfavorable for many biosynthetic and bioenergetic processes, and can lead to developmental disorders^26^.

### Disorders of amino acid metabolism in response to H-BPA exposure

Significant changes in amino acid metabolism were clearly observed in response to H-BPA exposure (Fig. 2b, Supplementary Figs S7 and S8). In alanine, aspartate and glutamate metabolism, significant reductions in glutamate, asparagine, aspartate and N-acetyl-aspartate were detected in response to embryonic exposure to TBBPA and TCBPA (Fig. 2b). Furthermore, glutamate, aspartate and N-acetyl-aspartate were also reduced in offspring embryos following parental exposure to TBBPA and TCBPA, illustrating that the decrease in alanine, aspartate and glutamate metabolism induced by H-BPA exposure was also heritable (Fig. 2b). Decreases in glutamate and aspartate indicated that fewer carbon and nitrogen sources were supplied to the TCA cycle for the production of energy, amino acids and pyrimidines in response to H-BPA exposure.

**Figure 3:**
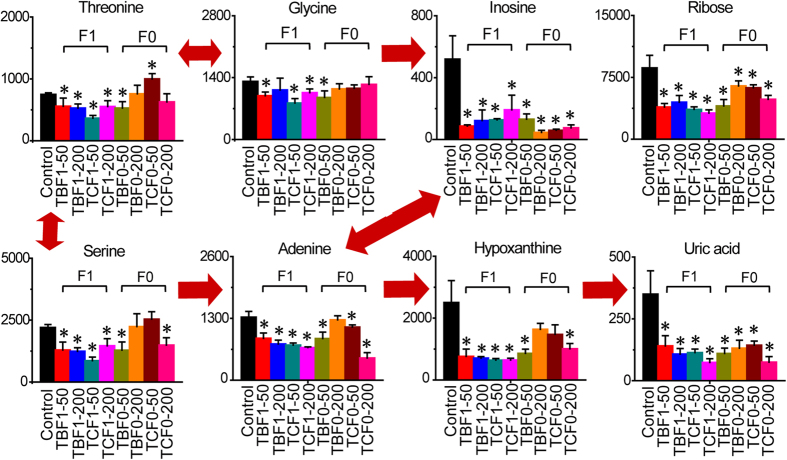
Disorders of nucleoside metabolism in response to H-BPA exposure. Metabolite levels are represented as the mean + sd. *p < 0.05, n = 4, two-tailed Mann-Whitney U test.

Large disruptions were also observed in the pathways of arginine and proline metabolism, aromatic and branched-chain amino acid metabolism, glycine, and serine and threonine metabolism after embryonic exposure to TBBPA and TCBPA (Supplementary Figs S7 and S8, Fig. 3). Citrulline, ornithine and proline were significantly reduced in H-BPA-exposed embryos; however, significant alterations in citrulline, ornithine and proline were not observed in offspring embryos follwoing parental exposure to H-BPA (Supplementary Fig. S7). Analysis of aromatic and branched-chain amino acid metabolism showed that valine, leucine, isoleucine, tyrosine, phenylalanine and tryptophan were all significantly decreased in H-BPA-exposed embryos, but the hereditary effects of the alterations in aromatic and branched-chain amino acids due to H-BPA exposure were not significant, excluding the effect on phenylalanine (Supplementary Fig. S8). The disruptions in aromatic and branched-chain amino acid metabolism revealed metabolic dysfunction in the livers of embryos exposed to H-BPA^27^. Additionally, the decreases in aromatic amino acids likely indicated that more precursors were utilized for the synthesis of hormones and neurotransmitters in response to H-BPA exposure. Moreover, glycine, serine and threonine metabolism was also significantly altered by H-BPA exposure, which is discussed in the section on nucleoside metabolism (Fig. 3).

### Disorders of nucleoside metabolism in response to H-BPA exposure

Nucleoside metabolism was significantly disturbed in embryos exposed to TBBPA and TCBPA (Fig. 3). Ribose, inosine, adenine, hypoxanthine and uric acid were all significantly reduced in H-BPA-exposed embryos. Moreover, reductions in ribose, inosine, adenine and uric acid were also observed in offspring embryos in response to parental exposure to H-BPA, illustrating that the H-BPA-induced changes in nucleoside metabolism were heritable. Moreover, glycine, serine and threonine were also significantly reduced in H-BPA-exposed embryos, indicating that fewer one-carbon sources were available for purine synthesis. The reduced nucleoside synthesis illustrated a delay in cell growth due to H-BPA exposure.

### Disorders of lipid metabolism in response to H-BPA exposure

Glycerol, glycerol-3-phosphate, o-phosphoethanolamine and pantothenic acid were significantly reduced in embryos exposed to TBBPA and TCBPA (Supplementary Fig. S9). Moreover, significant decreases in glycerol, glycerol-3-phosphate, o-phosphoethanolamine and pantothenic acid were also observed in offspring embryos following parental exposure to H-BPA, demonstrating that changes in these metabolites were heritable. Decreases in glycerol, glycerol-3-phosphate and o-phosphoethanolamine indicated that fewer building blocks for glycerophospholipid and glycerolipid were present in H-BPA-exposed embryos. Additionally, the reduction in pantothenic acid indicated a dearth of materials for the synthesis of coenzyme A, which participates in lipid and protein metabolism, following H-BPA exposure. Moreover, TBBPA was found to reduce lipid accumulation via activating oxidative pathways in rat hepatoma cells^28^. These findings demonstrated that lipid synthesis was inhibited by H-BPA exposure, and this inhibitory effect was heritable.

### Disorders of nervous system in response to H-BPA exposure

**Figure 4:**
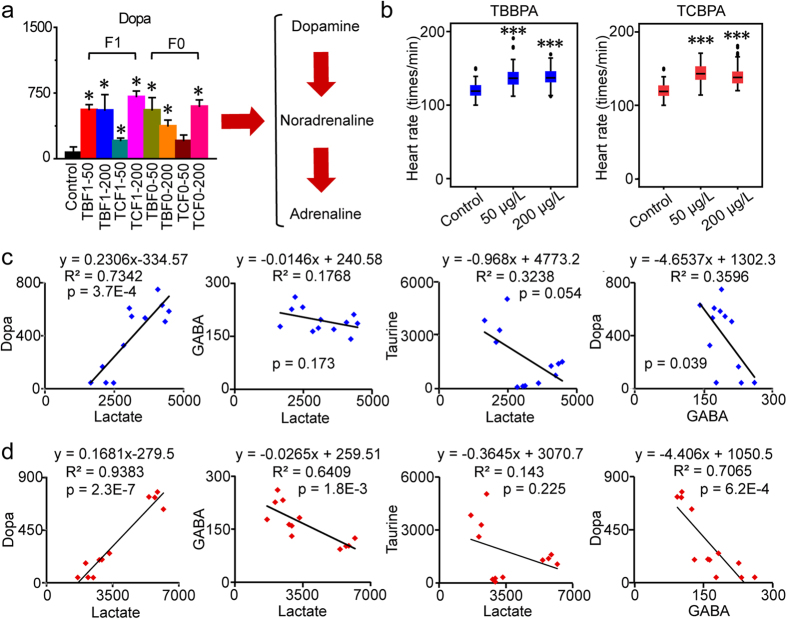
Disorders of neural system in response to H-BPA exposure. (**a**) Disorders of the dopamine pathway in response to H-BPA exposure. Dopa levels are represented as the mean + sd. *p < 0.05, n = 4, two-tailed Mann-Whitney U test. (**b**) Disorders of heart rates in response to H-BPA exposure. *p < 0.05; ***p < 0.001, n = 67–80, two-tailed Mann-Whitney U test. (**c**) Associations between neurotransmitters and lactate in response to TBBPA exposure. Data are from the control and treatment groups (n = 12). (**d**) Associations between neurotransmitters and lactate in response to TCBPA exposure. Data are from the control and treatment groups (n = 12).

Dopa levels increased significantly after embryonic exposure to TBBPA and TCBPA (Fig. 4a). A significant increase in dopa was also detected in offspring embryos following parental exposure to H-BPA, indicating that the disturbances in dopa were heritable (Fig. 4a). Dopa can be utilized directly for the synthesis of dopamine, noradrenaline and adrenaline. Decreases in phenylalanine and tyrosine and the increase in dopa supported an accelerated utilization of aromatic amino acids for the synthesis of catecholamines via the dopamine pathway, which has vital roles in the regulation of nerve and heart functions, such as the promotion of anxiety, fear, myocardial contractility and heart rate^29^,^30^. To further investigate alterations in cardiac functions and motor and neural behaviors induced by catecholamines due to H-BPA exposure, heart rates were examined. As illustrated, heart rates were significantly accelerated in response to TBBPA and TCBPA exposure at 50 and 200 μg/L, respectively, which was consistent with the increase in dopa levels (Fig. 4b).

To confirm the neural activation suggested by the increased levels of catecholamines in response to H-BPA exposure, alterations in taurine and 4-aminobutanoate (GABA), two major inhibitory neurotransmitters, were also assessed. We found that taurine was significantly decreased in embryos exposed to TBBPA and TCBPA, respectively, and that GABA was significantly reduced in offspring embryos after parental exposure to TBBPA and TCBPA (Supplementary Fig. S10). Taurine, which is one of major amino acids in the brain and is essential for normal development of the nervous system, stimulates GABA synthesis by activating GABA receptors and glutamate decarboxylase^31^,^32^. Therefore, activation of the dopamine pathway and decreases in inhibitory neurotransmitters confirmed the neural activation and disrupted development of the nervous system in response to H-BPA exposure.

Owing to the vital roles of neurotransmitters and lactate in the nervous system, correlations between dopa, GABA, taurine and lactate were performed to determine the roles of neurotransmitters in lactate accumulation (Fig. 4c,d). The increase in dopa had a significant positive correlation with lactate accumulation (*p* = 3.7E-4, R^2^ = 0.7342), whereas no significant correlations between GABA, taurine and lactate were observed in response to TBBPA exposure (*p* > 0.05). Moreover, the positive correlation between the increase in dopa and lactate accumulation was also significant (*p* = 2.3E-7, R^2^ = 0.9383), whereas GABA had a significant negative correlation with lactate (*p* = 1.8E-3, R^2^ = 0.6409), and no significant correlation between lactate and taurine was observed in response to TCBPA exposure (*p* > 0.05). Although a significant correlation was observed between lactate and GABA, higher R^2^, slopes and lower *p* values were observed in the linear regression model of lactate and dopa. Therefore, dopa had a stronger correlation with lactate accumulation and neural activation than GABA in response to H-BPA exposure. Moreover, significant correlations between dopa and GABA were observed in response to TBBPA and TCBPA exposure. The coordinated accumulation and reduction of dopa and GABA, respectively, led to neural activation accompanied by lactate accumulation, and the lactate accumulation in turn aggravated the neural disruptions in response to H-BPA exposure.

### Potential biomarkers for H-BPA exposure

**Figure 5:**
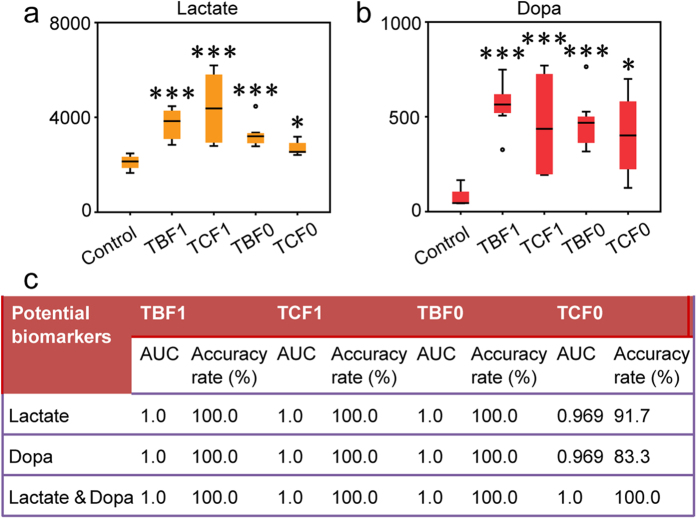
Potential biomarkers for H-BPA exposure. (**a**) Changes in lactate in response to H-BPA exposure. (**b**) Changes in dopa in response to H-BPA exposure. (**c**) Diagnostic performances of lactate and dopa in H-BPA exposure. *p < 0.05; ***p < 0.001, n = 4 and 8 in the control and treatment groups, respectively, two-tailed Mann-Whitney U test. TBF1: F0 was not exposed to H-BPA, whereas F1 was exposed to 50 and 200 μg/L TBBPA, respectively; TCF1: F0 was not exposed to H-BPA, whereas F1 was exposed to 50 and 200 μg/L TCBPA, respectively; TBF0: F0 was exposed to 50 and 200 μg/L TBBPA, respectively, whereas F1 was not exposed to H-BPA; TCF0: F0 was exposed to 50 and 200 μg/L TCBPA, respectively, whereas F1 was not exposed to H-BPA.

Lactate and dopa were significantly up-regulated in TBBPA and TCBPA-exposed embryos (Figs 1, 4a and 5). Notably, increases in lactate and dopa in offspring embryos also occurred in response to parental exposure to TBBPA and TCBPA, respectively (Figs 1, 4a and 5). The alterations in lactate and dopa were clearly indicative of the exposure to H-BPA and the consequent genetic effects. Accordingly, lactate and dopa were further employed to evaluate the effects of H-BPA exposure and related genetic effects using binary logistic regression.

Satisfactory diagnostic results for H-BPA exposure and related genetic effects were obtained using dopa and lactate either alone or in combination as diagnostic variables (Fig. 5c). As demonstrated, the effects of embryonic exposure to TBBPA and TCBPA, respectively, were accurately discriminated using lactate and dopa, either alone or in combination, as diagnostic variables; the diagnostic accuracy rates and AUC (area under the receiver operating characteristic curve) were 100.0% and 1.0, respectively. Additionally, when lactate and dopa were used as diagnostic variables, either alone or in combination, 100% of the embryos for which the parental embryos were exposed to TBBPA were accurately identified, providing an AUC of 1.0. Moreover, 91.7 and 83.3% of the embryos for which the parental embryos were exposed to TCBPA were also satisfactorily distinguished, with an AUC of 0.969 and 0.969 when lactate and dopa were employed alone as the diagnostic variable, respectively. Furthermore, an accuracy rate of 100% and an AUC of 1.0 were achieved for the identification of embryos for which the parental embryos were exposed to TCBPA when the combination of lactate and dopa was used as the diagnostic variable. Consequently, lactate and dopa, which are closely associated with neural activity, can be used as potential biomarkers to evaluate the effects of H-BPA exposure and related genetic effects. However, the practical utility of dopa and lactate as potential biomarkers for H-BPA exposure still needs large sample validation.

## Discussion

**Figure 6:**
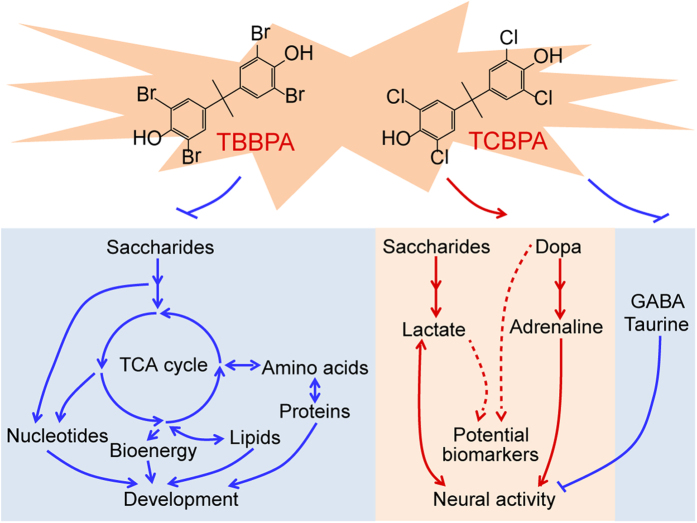
Schematic diagram of the major metabolic disorders associated with the molecular toxicology of H-BPA.

As discussed above, embryonic exposure to TBBPA and TCBPA significantly inhibited the synthesis of nucleosides, amino acids and lipids, as well as the supply of energy from the TCA cycle, thus interfering with normal organ development and suppressing developmental processes (Fig. 6). Unexpectedly, we observed an increase in lactate accumulation and dopamine pathway activation and a decrease in inhibitory neurotransmitters, together leading to neural activation in response to TBBPA and TCBPA exposure (Fig. 6). Notably, major metabolic disorders associated with the developmental toxicology of H-BPA (e.g., increases in neural activity and glycolysis, and decreases in the TCA cycle, glutamate and asparate metabolism, and lipid and nucleoside synthesis) were heritable. Furthermore, lactate and dopa, which are closely associated with neural activity, were identified as potential biomarkers for evaluating the effects of H-BPA exposure and related genetic effects.

We discovered a significant increase in the levels of dopa in H-BPA-exposed embryos. Increased dopa promotes the synthesis and release of catecholamines via the dopamine pathway, which have vital biological roles such as the modulation of neural activity, motor behaviors, neurogenesis, and cardiovascular functions, further affecting embryonic development^33^,^34^,^35^,^36^,^37^.

Uptake of dopamine, glutamate and GABA into synaptosomes and of dopamine into synaptic vesicles was reduced in response to TBBPA exposure^38^. Decreased synaptic and vesicular uptake of dopamine indicated a disruption of cytoplasmic dopamine utilization during the synthesis of noradrenaline and adrenaline. In addition, a decrease in GABA and taurine and an increase in dopa were observed in response to TBBPA and TCBPA exposure. The coordinated alterations between catecholamines and inhibitory neurotransmitters resulted in neural activation accompanied by increased lactate accumulation and heart rates in response to H-BPA exposure. Moreover, in correlation analysis, we shown that catecholamines had stronger correlations with lactate accumulation than GABA in in response to H-BPA exposure.

Previous evidence has demonstrated that neural activation induces lactate accumulation in the brains of patients with panic disorders, and lactate-induced panics can be blocked in patients who are treated with monoamine oxidase inhibitors, suggesting a vital role of catecholamines in lactate-induced anxiety symptoms^24^,^39^,^40^,^41^. Additionally, heart rates and other anxiety symptoms are significantly increased in rats treated with chronic GABA receptor blockade in response to lactate infusion, and panic-like responses to lactate can be blocked by GABAergic tone restoration, revealing important roles of GABA in lactate-induced anxiety and panic^40^,^42^.

Moreover, the reduced dopamine uptake into synaptosomes suggestes a greater accumulation of plasma dopamine, potentially activating dopamine receptor D2 (DRD2) signaling. DRD2 activation has been shown to greatly decrease AKT activity by suppressing the phosphorylation by forming a complex with PP2A and β-arrestin, and DRD2-dependent decreases in AKT signaling lead to a significant reduction of the GABAergic neural population in the brain and disrupted motor behaviors in zebrafish larvae^34^,^43^. Notably, AKT signaling can modulate cellular proliferation, differentiation and organ development via its downstream targets, including mTOR and GSK3β, thus influencing embryonic development^34^,^44^,^45^,^46^. Taken together, the morphological lesions and developmental retardation observed in H-BPA-exposed embryos was probably associated with DRD2-dependent reductions in AKT signaling, warranting further investigations.

In conclusion, to the best of our knowledge, this is the first comprehensive landscape of metabolic disorders associated with the molecular toxicology of TBBPA and TCBPA. The results showed that decreases in the synthesis of nucleosides, amino acids and lipids, a disrupted energy supply from the TCA cycle and glycolysis, and neural activation were related to the developmental retardation following H-BPA exposure. Moreover, dopa and lactate were found to be potential biomarkers for assessing the developmental toxicity of H-BPA exposure and related genetic effects. The metabolomics approach used in this study was found to be an useful tool for elucidating molecular mechanisms, regulatory targets and potential biomarkers in toxicology and risk assessment studies of environmental pollutants.

## Methods

### Chemicals and reagents

TBBPA (97%) and TCBPA (98%) were obtained from Tokyo Chemical Industry (Tokyo, Japan). Ultrapure water was prepared with the Milli-Q system (Millipore Corp., Billerica, MA, USA), and HPLC-grade methanol was obtained from Honeywell Burdick & Jackson (Muskegon, MI, USA). Dimethyl sulfoxide (DMSO, ≥99.5%), tridecanoic acid (≥98%), methoxyamine hydrochloride (98%), pyridine (anhydrous, 99.8%) and N-methyl-N-(trimethylsilyl)-trifluoroacetamide (MSTFA, for GC derivation, ≥98.5%) were all purchased from Sigma-Aldrich (Shanghai, China).

### Embryonic exposure experiments and sample collections

All animal protocols used in this study were approved by the Institutional Animal Ethics Committee of Institute of Urban Environment, Chinese Academy of Sciences, and performed in compliance with the guidelines of Institutional Animal Care and Use Committee, and the European Communities Directive (86/609/EEC). Six-month-old O. melastigma fish were cultured in 28 ± 1 °C artificial seawater at 3% salinity. The fish were fed live brine shrimp nauplii (Artemia sp.) twice per day under a 14:10 light-dark cycle. Synchronized fertilized embryos were obtained within 2 hours after the initiation of photophase, and fertilization was confirmed using a dissecting microscope.

To assess the developmental toxicity of H-BPA, O. melastigma embryos were exposed to TBBPA and TCBPA (0, 50, 200 and 800 μg/L) from 2 to 10 dpf (days post fertilization) according to environmentally relevant concentrations and the criteria for aquatic life based on the guidelines of U.S. Environmental Protection Agency^12^,^47^. TBBPA and TCBPA were first dissolved in DMSO to prepare the stock solution, and the final DMSO concentration in artificial seawater was 0.2% in each group. For each replicate, 35 embryos were randomly selected and cultured in 90-mm Petri dishes with 20-mL artificial seawater to evaluate the effects of H-BPA exposure on morphology and hatchability. Three replicates were conducted for each group, and the culture liquid was refreshed daily. Newly hatched larvae were transferred to a 300-mL glass break added with 150-mL artificial seawater, which was also refreshed daily. At 10 dpf, embryos without heart rates nor blood circulation were identified as dead, and the hatchability was determined^48^. Detailed methods for measuring heart rates have been described in our previous work^49^. Briefly, the heart beats of embryos were directly counted for 60 s using a dissecting microscope after collection from the dishes.

To further elucidate the molecular developmental toxicology of H-BPA, the offspring embryos, the parental embryos of which were not exposed to H-BPA, were treated with 0, 50 and 200 μg/L TBBPA and TCBPA, respectively, from 2 to 6 dpf. Furthermore, to determine whether the developmental toxicity of H-BPA was heritable, parental embryos were exposed to 0, 50 and 200 μg/L TBBPA and TCBPA, respectively, from 2 to 6 dpf, whereas offspring embryos did not receive H-BPA treatment. Subsequently, offspring embryos at 6 dpf were collected for the metabolomics analysis, and 4 replicates were evaluated in each group.

According to the H-BPA treatment, embryo samples collected for metabolomics analysis were further classified into 9 types: Control (neither F0 nor F1 exposed to H-BPA), TBF1–50 (F0, not exposed to H-BPA; F1, exposed to 50 μg/L TBBPA), TBF1–200 (F0, not exposed to H-BPA; F1, exposed to 200 μg/L TBBPA), TCF1–50 (F0, not exposed to H-BPA; F1, exposed to 50 μg/L TCBPA), TCF1-200 (F0, not exposed to H-BPA; F1, exposed to 200 μg/L TCBPA), TBF0-50 (F0, exposed to 50 μg/L TBBPA; F1, not exposed to H-BPA), TBF0-200 (F0, exposed to 200 μg/L TBBPA; F1, not exposed to H-BPA), TCF0-50 (F0, exposed to 50 μg/L TCBPA; F1, not exposed to H-BPA) and TCF0-200 (F0, exposed to 200 μg/L TCBPA; F1, not exposed to H-BPA). Additional information regarding embryonic exposure experiments and sample collections is listed in Supplementary Fig. S1.

### Sample preparation

Twelve offspring embryos in each replicate were accurately weighted and transferred to an Eppendorf tube, to which were added steel balls and 600-μL methanol/water solution (v/v = 4:1, 5 μg/mL tridecanoic acid as the internal standard). Subsequently, the embryos were homogenized at 33 times/s for 1.5 min; and centrifuged at 12000 rpm at 4 °C for 15 min. After centrifugation, 480 μL supernatant was vacuum-dried in a Speedvac concentrator (Thermo Scientific, USA). Fifty microliters of methoxyamine solution (20 mg/mL in pyridine) was added to the dried sample, and vortexed for 30 s. The sample was then placed in a 37 °C water bath for 1.5 h of oximation reaction, followed by silylation reaction with 40 μL MSTFA in a 37 °C water bath for 1 h. Ultimately, the derivatized sample was centrifuged at 12000 rpm at 4 °C for 15 min, and the supernatant was collected for subsequent GC-MS analysis.

The residual supernatant in each sample was collected and mixed for 5 min to prepare the QC (quality control) samples. One QC sample was inserted between each set of 10 analytical samples; and processed equivalently during vacuum drying, derivatization and subsequent GC-MS analysis to evaluate the reproducibility and stability of metabolomics approach.

### GC-MS analysis

Metabolic profiling of the sample was obtained by employing GC-MS (GCMS-QP 2010 plus, Shimadzu, Japan) equipped with an AOC-20i autosampler, into which was injected 1 μL derivatized sample. A DB-5 MS capillary column (30 m × 250 μm × 0.25 μm, J&W Scientific Inc., USA) was used for the metabolite separations. Helium (carrier gas) passed through the column at a constant linear velocity of 40.0 cm/s, and the split ratio was 10. The initial oven temperature was maintained at 70 °C for 3.0 min, increased to 300 °C at a rate of 5 °C/min, and ultimately maintained for 10 min. The temperatures of the inlet, interface and ion source were 300, 280 and 230 °C, respectively. EI (electron impact, 70 eV) was used as the ionization mode, and the detector voltage was set according to the tuning result. Mass signals were acquired in full scan mode (m/z, 33–600). The solvent delay time and even time were 5.3 min and 0.2 s, respectively. Retention times of n-alkanes in a light diesel sample were acquired to calculate the retention indexes of the metabolites after running all samples.

### Data processing

Raw MS data was converted into the NetCDF format using GCMS solution 2.7 (Shimadzu, Japan); and then used for peak matching employing XCMS^50^. After deconvolution of the MS signals using ChromaTOF 4.43 (LECO Corporation, USA), feature ions of metabolites those were omitted during the process of peak matching were added to the peak table, ultimately resulting in the inclusion of 2860 ions in the peak table. Metabolites were identified mainly based on the results of mass spectra searches of commercial libraries (i.e., NIST 11, Fiehn and Wiley); and further confirmed according to available reference standards based on the mass spectra, retention time and retention index. The raw peak area of metabolites in the peak table were normalized to that of tridecanoic acid and the tissue weight; and then multiplied by 1×10^5^. The data were then applied for subsequent statistical analysis. A nonparametric test (two-tailed Mann-Whitney U test) was performed using MATLAB 7.11 (MathWorks, USA) to assess the differences between the control and treatment groups with regard to hatchability, hatchability at 7 dpf, levels of metabolites and heart rates. The significance level was 0.05. PCA (principal component analysis), PLS-DA (partial least-squares discriminant analysis) and pathway analysis were performed using MetaboAnalyst 3.0^51^. In the process of pathway analysis, levels of metabolites were mean-centered and divided by the standard deviation of each metabolite to make the data obey the Gaussian distribution. After the zebra fish pathway library was selected, Global Test and Relative-betweeness Centrality were subsequently employed as the algorithms for pathway enrichment analysis and topology analysis, respectively. Besides, all metabolites in the zebra fish pathways were used as the reference metabolome. The heat map plot was generated using MeV 4.9.0^52^.

## Additional Information

**How to cite this article**: Ye, G. *et al*. Metabolomics approach reveals metabolic disorders and potential biomarkers associated with the developmental toxicity of tetrabromobisphenol A and tetrachlorobisphenol A. *Sci. Rep.* **6**, 35257; doi: 10.1038/srep35257 (2016).

## Supplementary Material

Supplementary Information

Supplementary Table S1
