# Supplementary material for: Metabolomics approach reveals metabolic disorders and potential biomarkers associated with the developmental toxicity of tetrabromobisphenol A and tetrachlorobisphenol A
Source: Sci Rep. 2016 Oct 13;6:35257. doi: 10.1038/srep35257 (PMC5062249; doi:10.1038/srep35257)
Supplement: Supplementary Information [file srep35257-s1.pdf]

Supplementary information for

Metabolomics approach reveals metabolic disorders and potential biomarkers associated with the developmental toxicity of tetrabromobisphenol A and tetrachlorobisphenol A

Guozhu Ye[1,#], Yajie Chen[1,2,#], Hong-ou Wang[1,2,#], Ting Ye[1,2], Yi Lin[1], Qiansheng Huang[1], Yulang Chi[1], Sijun Dong[1,*]

[1]Key Laboratory of Urban Environment and Health, Institute of Urban Environment, Chinese Academy of Sciences, Xiamen 361021, China

[2]University of Chinese Academy of Sciences, Beijing 100049 , China

[*]Corresponding Authors

Tel: +86-592-6190-779, fax: +86-592-6190-566, e-mail: sjdong@iue.ac.cn

[#]These authors contributed equally to this work.

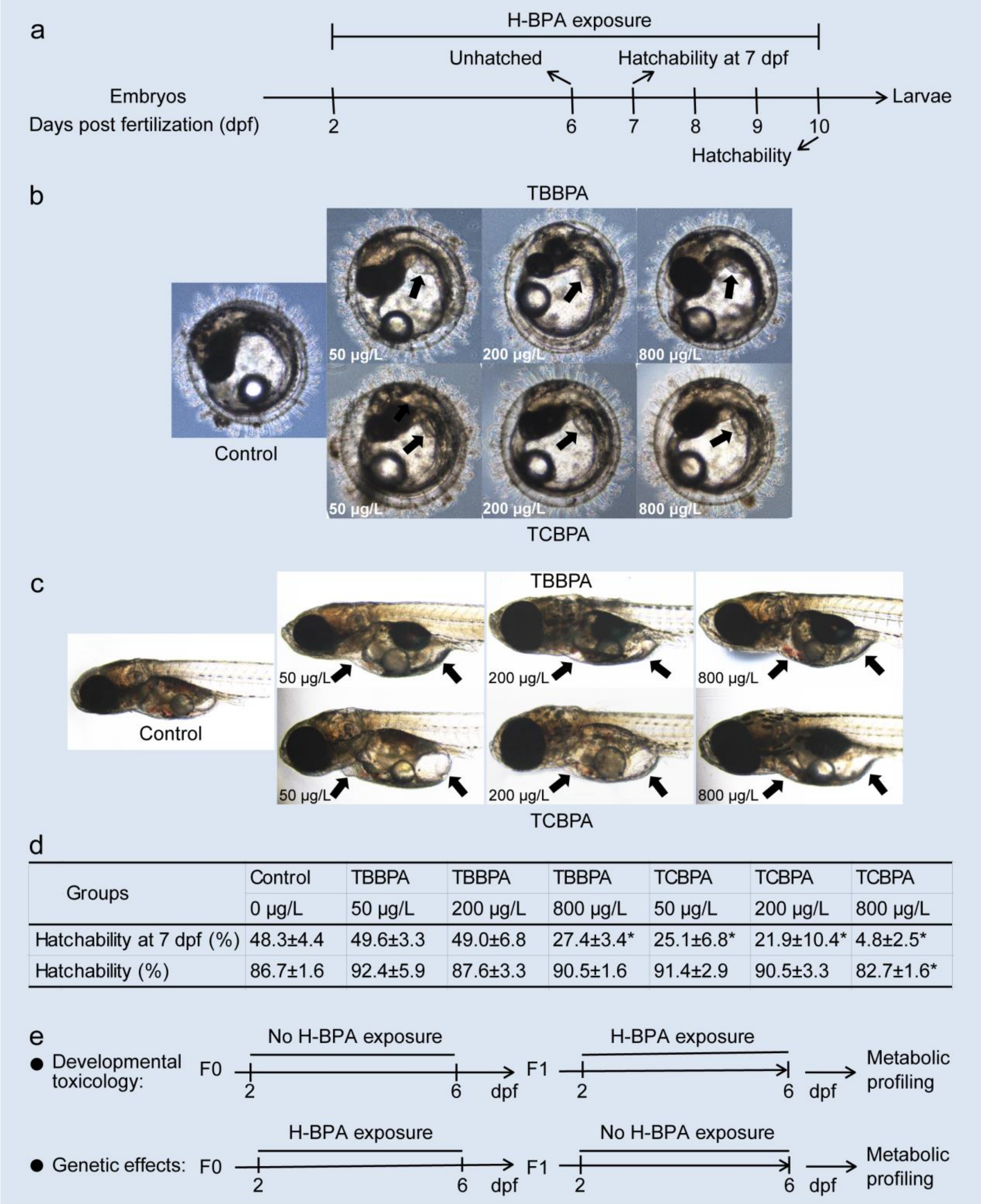

Groups	Control	TBBPA	TBBPA	TBBPA	TCBPA	TCBPA	TCBPA
	0 μg/L	50 μg/L	200 μg/L	800 μg/L	50 μg/L	200 μg/L	800 μg/L
Hatchability at 7 dpf (%)	48.3±4.4	49.6±3.3	49.0±6.8	27.4±3.4*	25.1±6.8*	21.9±10.4*	4.8±2.5*
Hatchability (%)	86.7±1.6	92.4±5.9	87.6±3.3	90.5±1.6	91.4±2.9	90.5±3.3	82.7±1.6*

Figure S1. Experimental designs and morphological changes related to the developmental toxicity of H-BPA in O. melastigma embryos. (**a**) Experimental designs for embryonic exposure to H-BPA. (**b**) Morphological changes in embryos in response to H-BPA exposure. (**c**) Morphological changes in larvae in response to H-BPA exposure. (**d**) Changes in the hatchability in response to H-BPA exposure. (**e**) Experimental designs for discovering the developmental toxicology and related genetic effects of H-BPA using the metabolomics analysis.

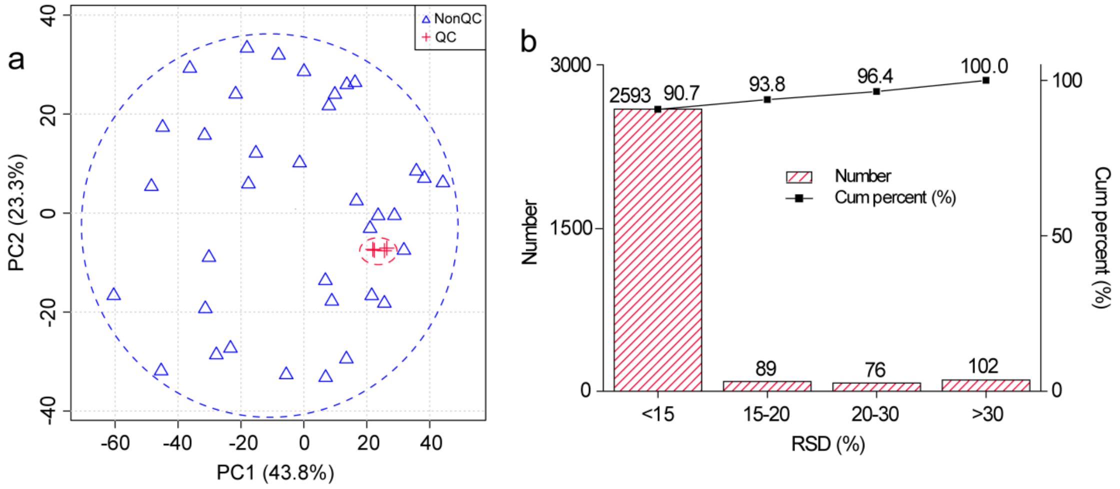

Figure S2. Analytical performance of metabolic profiling. (**a**) PCA score plot of the samples. (**b**) Distribution of RSDs (relative standard deviations) of the metabolites in QC samples.

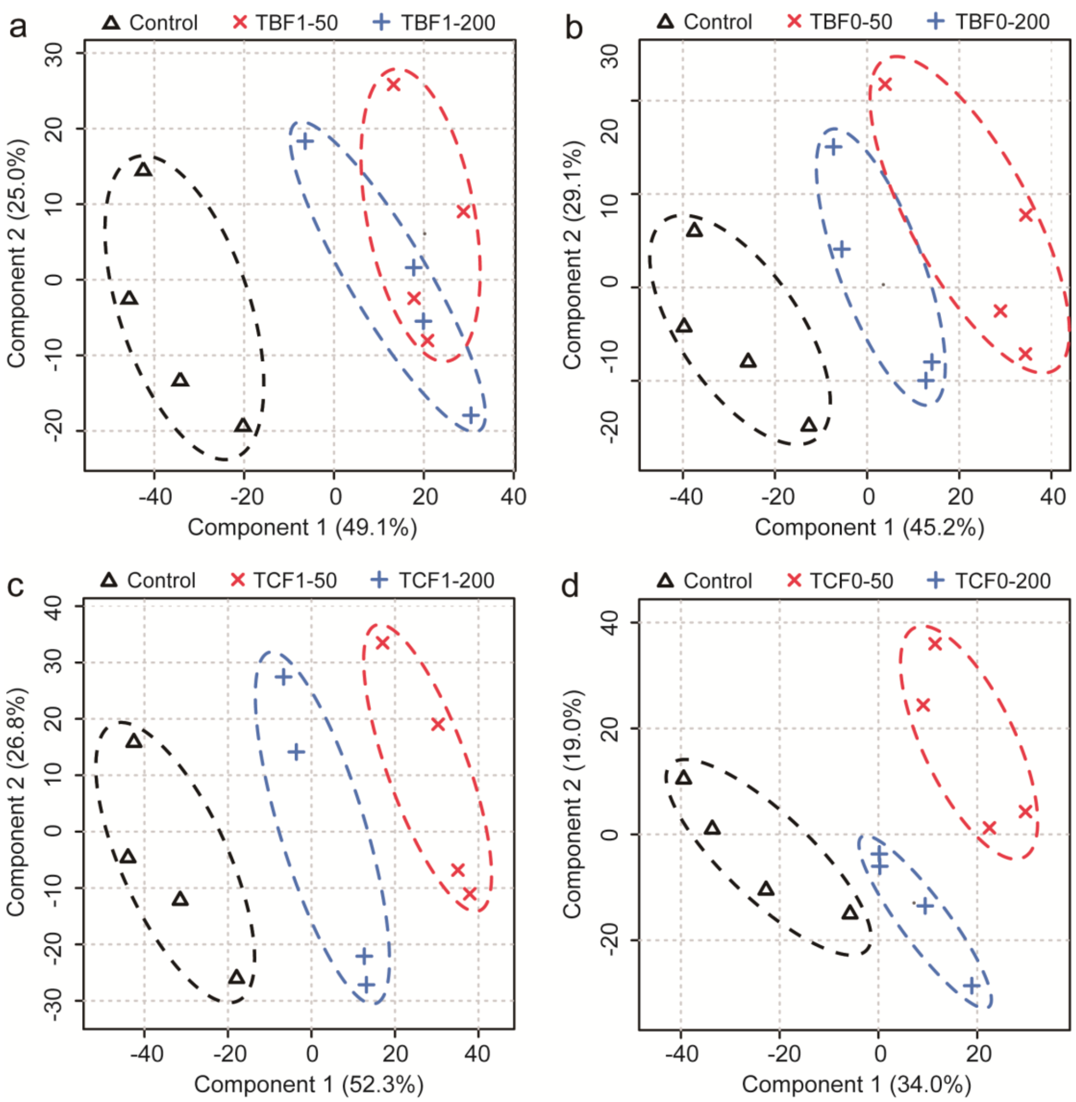

Figure S3. Evaluation of changes in metabolic profiling of O. melastigma embryos in response to H-BPA exposure using PLS-DA score plot. (**a**) Changes in metabolic profiling of TBF1 embryos (F0, not exposed to H-BPA; F1, exposed to 50 and 200 μg/L TBBPA, respectively). (**b**) Changes in metabolic profiling of TBF0 embryos (F0, exposed to 50 and 200 μg/L TBBPA, respectively; F1, not exposed to H-BPA). (**c**) Changes in metabolic profiling of TCF1 embryos (F0, not exposed to H-BPA; F1, exposed to 50 and 200 μg/L TCBPA, respectively). (**d**) Changes in metabolic profiling of TCF0 embryos (F0, exposed to 50 and 200 μg/L TCBPA, respectively; F1, not exposed to H-BPA).

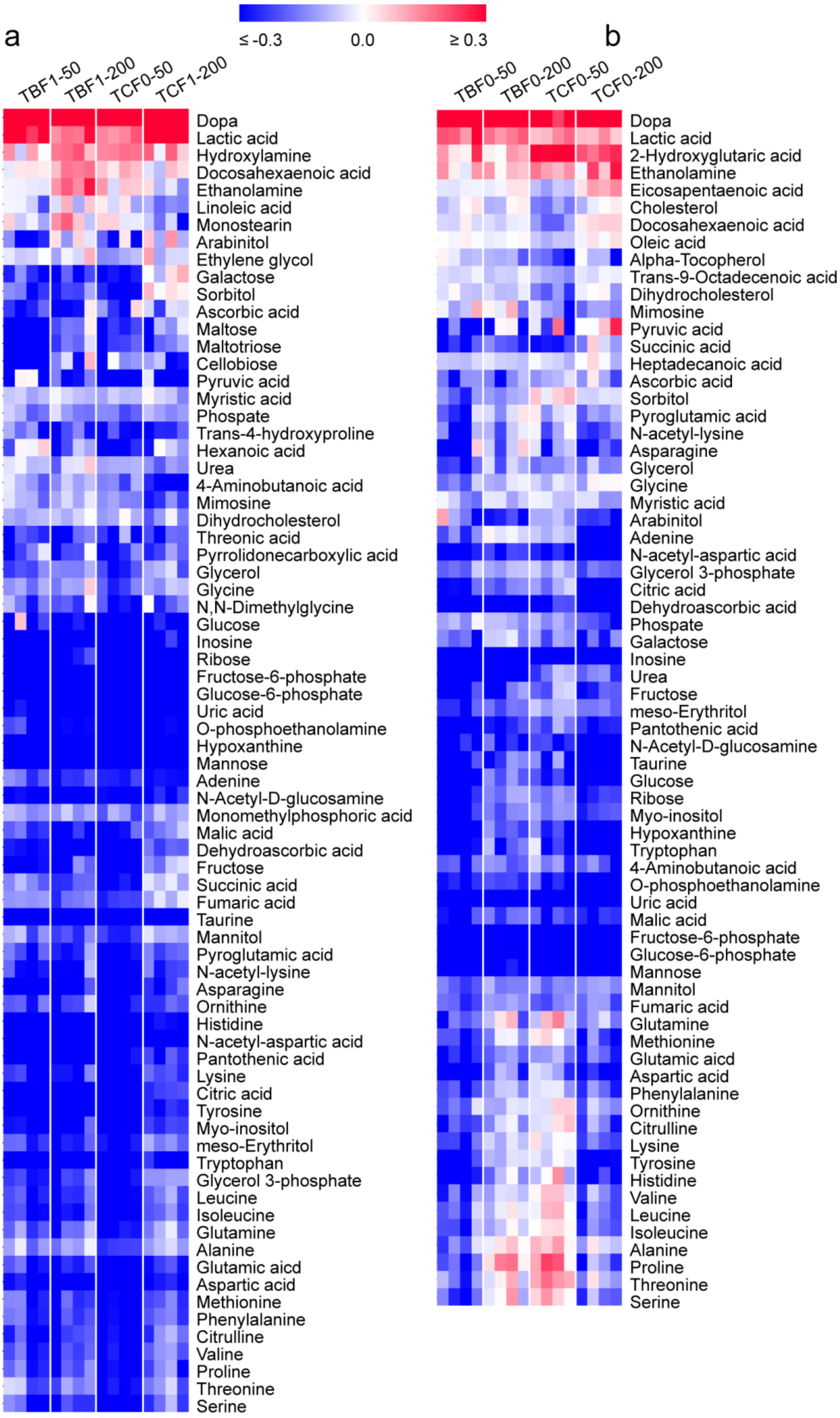

Figure S4. Changes in differential metabolites in response to H-BPA exposure using heat map. The differential metabolites had *p* values below 0.05 in at least one of the comparisons between the control

and treatment groups (n = 4, two-tailed Mann-Whitney U test). Ratios of the metabolite levels in the treatment groups to the average level in the control group were first obtained, and then changes in differential metabolites were represented as $\log_{10}^{(ratio)}$, which were used for heat map plot. Red color indicates that metabolite levels in treatment groups are higher than the average level in the control group, whereas blue color indicates that metabolite levels in the treatment groups are lower than the average level in the control group. (**a**) Metabolites significantly changed ($p < 0.05$) in offspring embryos resulting from TBBPA and TCBPA exposure. (**b**) Metabolites significantly changed ($p < 0.05$) in offspring embryos resulting from parental embryonic exposure to TBBPA and TCBPA.

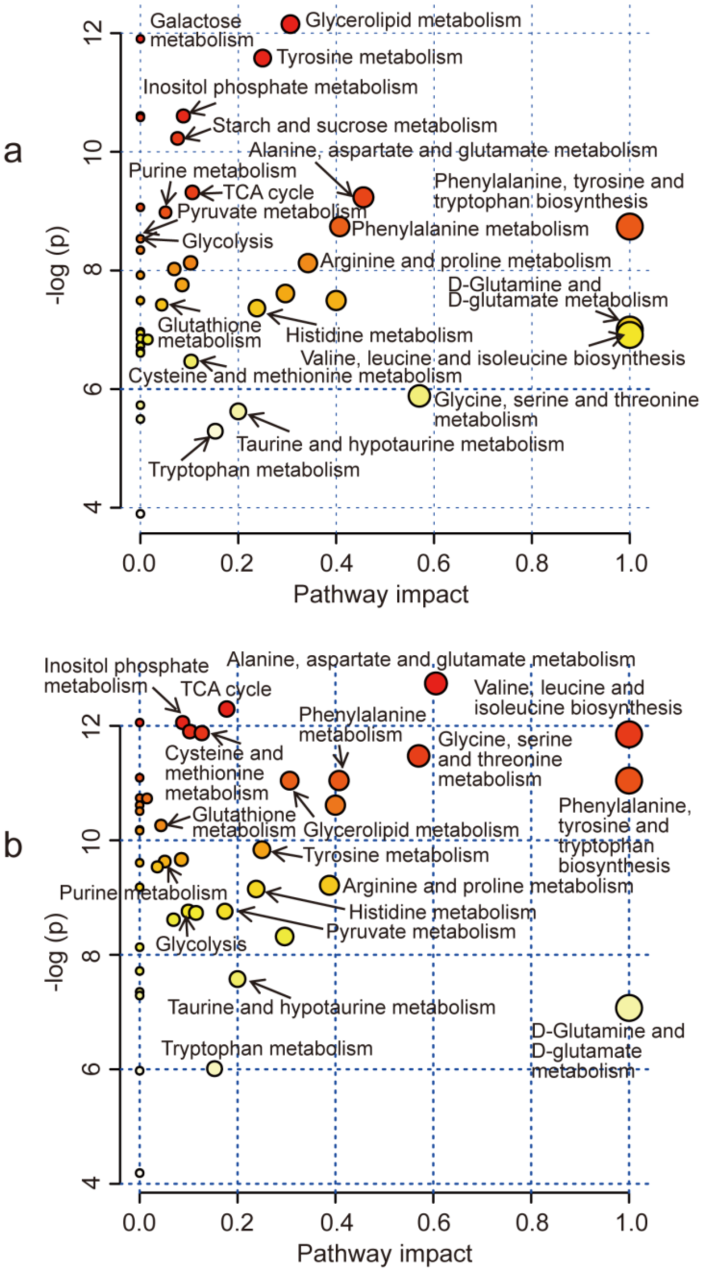

Figure S5. Disorders of metabolic pathways in TBF1-50 and TCF1-50 embryos in response to H-BPA exposure. (**a**) Changes in metabolic pathways in TBF1-50 embryos in response to TBBPA exposure. (**b**) Changes in metabolic pathways in TCF1-50 embryos in response to TCBPA exposure.

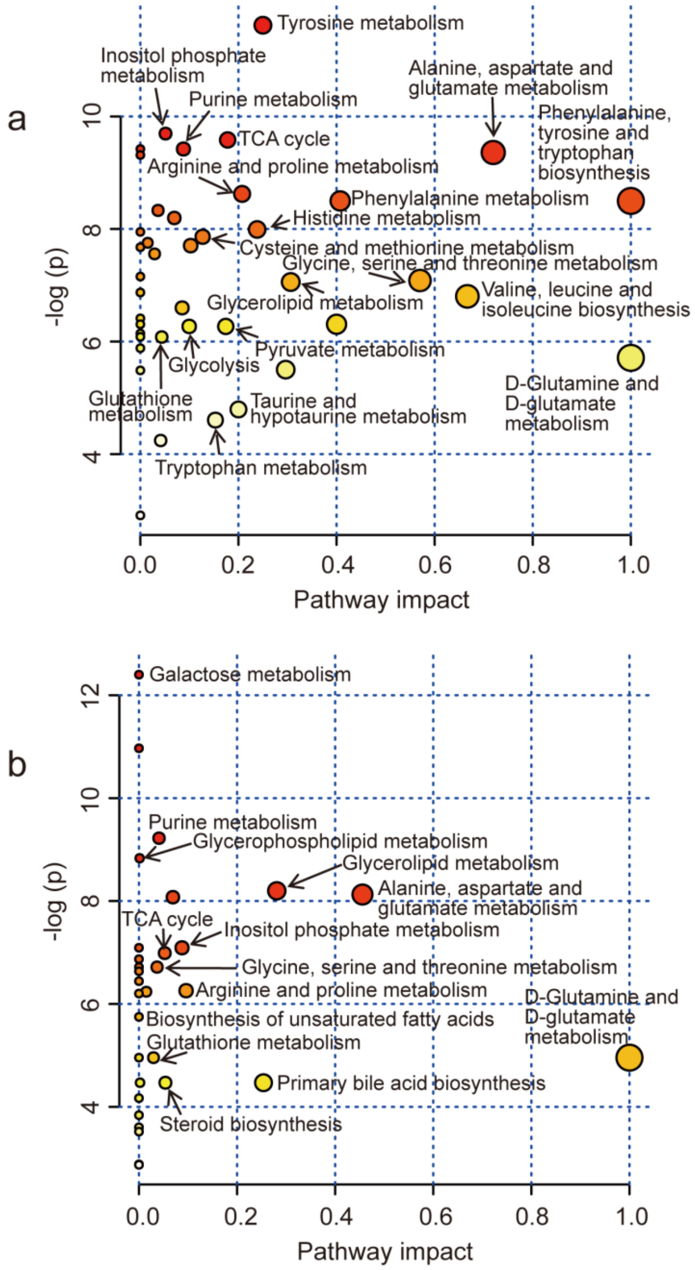

Figure S6. Disorders of metabolic pathways in TBF0-50 and TCF0-50 embryos in response to H-BPA exposure. (**a**) Changes in metabolic pathways in TBF0-50 embryos in response to TBBPA exposure. (**b**) Changes in metabolic pathways in TCF0-50 embryos in response to TCBPA exposure.

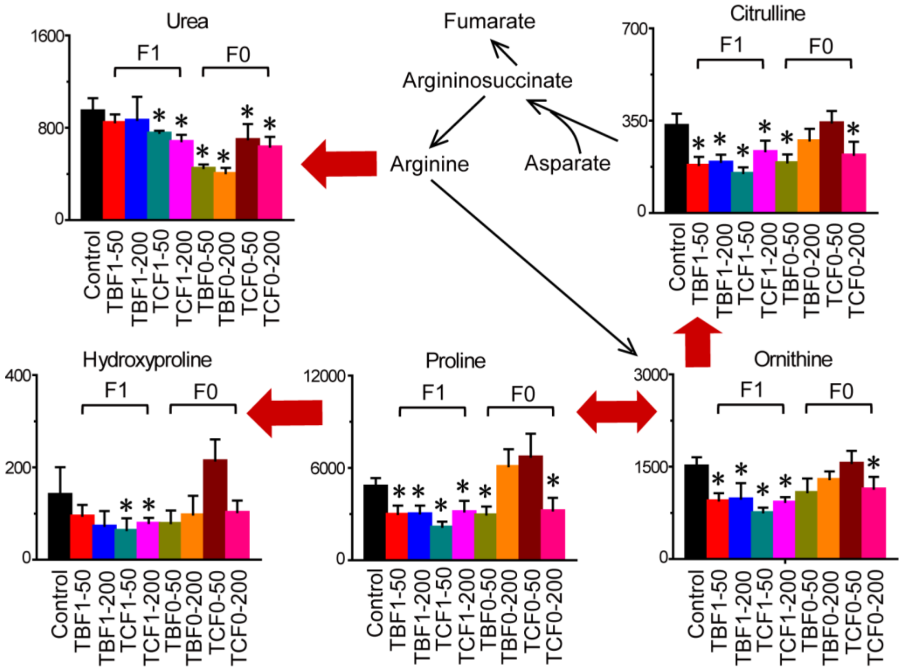

Figure S7. Disorders of arginine and proline metabolism in response to H-BPA exposure. Metabolite levels are represented as the mean + sd. *, $p < 0.05$, n = 4, two-tailed Mann-Whitney U test.

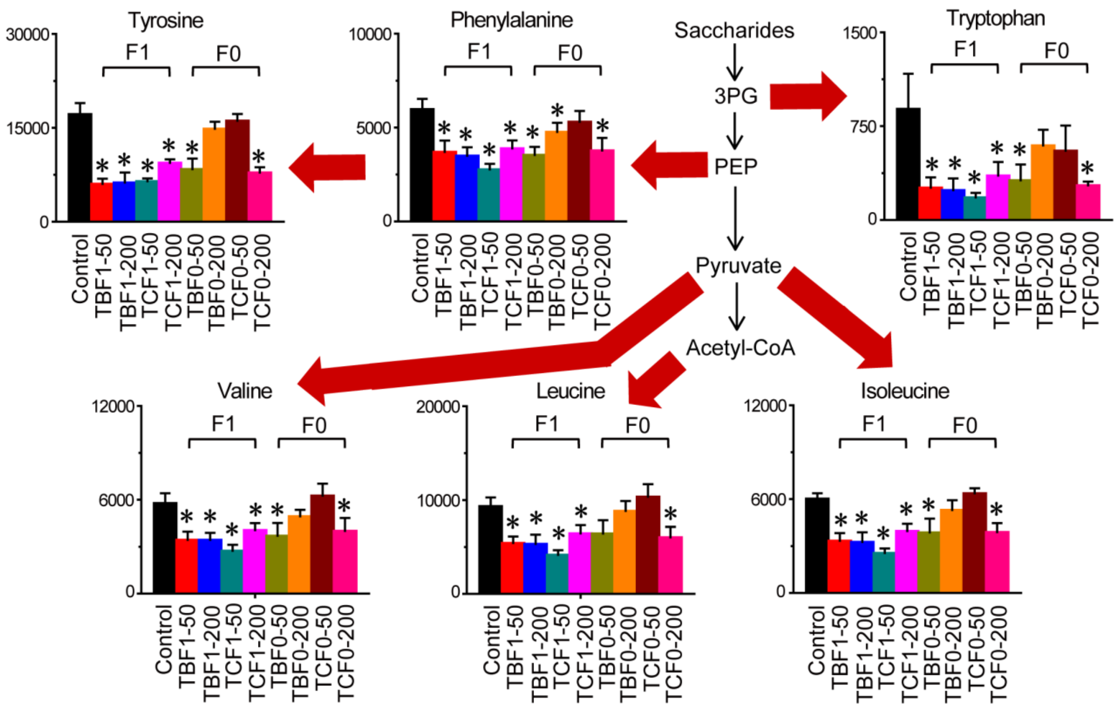

Figure S8. Disorders of aromatic and branched-chain amino acid metabolism in response to H-BPA exposure. Metabolite levels are represented as the mean + sd. *, $p < 0.05$, n = 4, two-tailed Mann-Whitney U test.

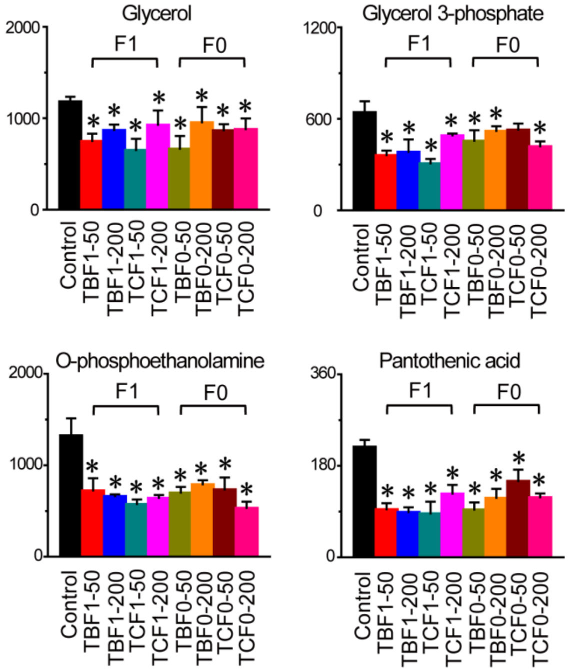

Figure S9. Disorders of lipid metabolism in response to H-BPA exposure. Metabolite levels are represented as the mean + sd. *, $p < 0.05$, n = 4, two-tailed Mann-Whitney U test.

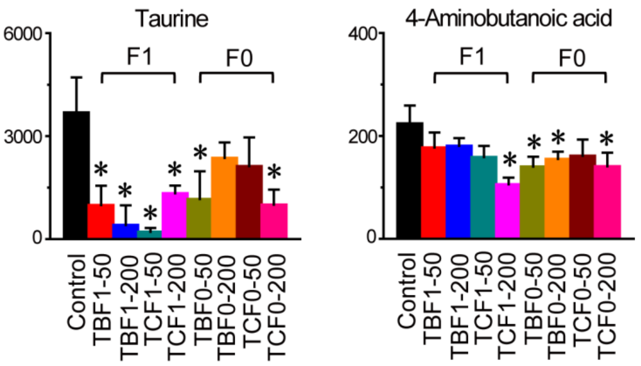

Figure S10. Disorders of inhibitory neurotransmitters in response to H-BPA exposure. Metabolite levels are represented as the mean + sd. *, $p < 0.05$, n = 4, two-tailed Mann-Whitney U test.

Table S1. Changes in the differential metabolites in response to H-BPA exposure.

Metabolites	TBF1-50/C[c]		TBF1-200/C		TCF1-50/C		TCF1-200/C		TBF0-50/C		TBF0-200/C		TCF0-50/C		TCF0-200/C	
	p	Ratio	*p*	Ratio	*p*	Ratio	*p*	Ratio	*p*	Ratio	*p*	Ratio	*p*	Ratio	*p*	Ratio
Carbohydrate metabolism																
Glucose[a]	0.486	0.60	0.057	0.46	**0.029**	**0.33**	0.057	0.47	**0.029**	**0.28**	0.686	0.69	0.343	0.57	**0.029**	**0.49**
Fructose-6-phosphate[a]	**0.029**	**0.31**	**0.029**	**0.38**	**0.029**	**0.30**	**0.029**	**0.29**	**0.029**	**0.28**	**0.029**	**0.39**	**0.029**	**0.27**	**0.029**	**0.25**
Glucose-6-phosphate[a]	**0.029**	**0.25**	**0.029**	**0.31**	**0.029**	**0.23**	**0.029**	**0.24**	**0.029**	**0.27**	**0.029**	**0.34**	**0.029**	**0.17**	**0.029**	**0.20**
Pyruvic acid[a]	**0.686**	**0.70**	**0.029**	**0.61**	**0.029**	**0.32**	0.114	0.35	**0.029**	**0.46**	0.686	0.70	0.686	0.83	0.200	1.25
Lactic acid[a]	**0.029**	**1.98**	**0.029**	**1.56**	**0.029**	**1.43**	**0.029**	**2.76**	**0.029**	**1.65**	**0.029**	**1.46**	**0.029**	**1.32**	0.057	1.24
Fructose[a]	**0.029**	**0.45**	**0.029**	**0.53**	**0.029**	**0.37**	0.114	0.80	**0.029**	**0.49**	**0.029**	**0.62**	0.114	0.76	**0.029**	**0.58**
Mannose[a]	**0.029**	**0.38**	**0.029**	**0.39**	**0.029**	**0.32**	**0.029**	**0.28**	**0.029**	**0.25**	**0.029**	**0.40**	**0.029**	**0.30**	**0.029**	**0.34**
Mannitol[a]	**0.029**	**0.75**	**0.029**	**0.62**	**0.029**	**0.57**	0.057	0.83	**0.029**	**0.64**	**0.029**	**0.73**	**0.029**	**0.72**	**0.029**	**0.75**
meso-Erythritol[a]	**0.029**	**0.61**	**0.029**	**0.55**	**0.029**	**0.51**	**0.029**	**0.73**	**0.029**	**0.57**	**0.029**	**0.66**	**0.029**	**0.82**	**0.029**	**0.68**
Arabinitol[a]	**0.029**	**0.56**	1.000	0.98	0.200	0.62	1.000	1.03	0.343	0.75	**0.029**	**0.54**	**0.029**	**0.80**	**0.029**	**0.56**
Maltose[a]	**0.029**	**0.38**	0.343	0.76	**0.029**	**0.55**	0.200	0.72	0.114	0.67	0.114	1.32	0.886	1.00	0.343	1.16
Cellobiose[a]	**0.029**	**0.32**	0.486	0.84	0.486	0.69	0.114	0.63	0.343	0.68	0.114	1.58	0.114	1.44	0.886	1.18
Galactose[a]	**0.029**	**0.59**	**0.029**	**0.52**	**0.029**	**0.50**	0.686	1.04	**0.029**	**0.63**	**0.029**	**0.85**	**0.029**	**0.73**	**0.029**	**0.62**
Sorbitol[a]	**0.029**	**0.63**	**0.029**	**0.63**	**0.029**	**0.50**	0.343	1.09	**0.029**	**0.67**	**0.029**	**0.77**	0.200	1.12	0.057	0.90
Ribose[a]	**0.029**	**0.45**	**0.029**	**0.52**	**0.029**	**0.42**	**0.029**	**0.37**	**0.029**	**0.47**	**0.029**	**0.75**	**0.029**	**0.72**	**0.029**	**0.56**
N-Acetyl-D-glucosamine[a]	**0.029**	**0.38**	**0.029**	**0.31**	**0.029**	**0.33**	**0.029**	**0.45**	**0.029**	**0.37**	**0.029**	**0.50**	**0.029**	**0.57**	**0.029**	**0.45**
Ascorbic acid[a]	**0.029**	**0.57**	0.114	0.60	0.200	0.73	0.200	0.82	**0.029**	**0.66**	0.057	0.69	0.200	0.82	0.200	0.80
Dehydroascorbic acid[a]	**0.029**	**0.52**	**0.029**	**0.36**	**0.029**	**0.44**	**0.029**	**0.65**	**0.029**	**0.40**	**0.029**	**0.25**	**0.029**	**0.52**	**0.029**	**0.14**
Threonic acid[a]	**0.029**	**0.53**	**0.029**	**0.55**	0.057	0.55	**0.029**	**0.60**	0.114	0.72	0.686	1.17	0.686	0.91	0.200	1.44
Myo-inositol[a]	**0.029**	**0.49**	**0.029**	**0.52**	**0.029**	**0.43**	**0.029**	**0.59**	**0.029**	**0.48**	**0.029**	**0.69**	**0.029**	**0.75**	**0.029**	**0.60**
Maltotriose[a]	**0.029**	**0.42**	0.057	0.71	**0.029**	**0.58**	0.114	0.65	0.057	0.58	0.886	1.01	0.686	0.96	0.200	0.75

Table S1 Continued.

Ethylene glycol[b]	0.486	0.92	0.886	0.97	**0.029**	**0.78**	0.686	0.97	0.486	0.93	1.000	1.02	0.486	1.09	0.114	1.16
TCA cycle																
Citric acid[a]	**0.029**	**0.35**	**0.029**	**0.33**	**0.029**	**0.29**	**0.029**	**0.60**	**0.029**	**0.52**	**0.029**	**0.68**	0.057	0.78	**0.029**	**0.30**
Fumaric acid[a]	**0.029**	**0.75**	**0.029**	**0.67**	**0.029**	**0.58**	0.114	0.86	**0.029**	**0.62**	**0.029**	**0.76**	**0.029**	**0.68**	**0.029**	**0.70**
Malic acid[a]	**0.029**	**0.62**	**0.029**	**0.49**	**0.029**	**0.54**	**0.029**	**0.74**	**0.029**	**0.53**	**0.029**	**0.66**	**0.029**	**0.59**	**0.029**	**0.56**
Succinic acid[a]	**0.029**	**0.73**	**0.029**	**0.62**	**0.029**	**0.47**	0.486	0.88	**0.029**	**0.55**	**0.029**	**0.63**	**0.029**	**0.55**	0.486	0.87
2-Hydroxyglutaric acid[a]	0.114	1.39	0.200	1.38	0.886	0.93	0.486	1.22	0.486	1.30	0.686	1.15	**0.029**	**2.13**	**0.029**	**1.63**
Amino acid metabolism																
Glycine[a]	**0.029**	**0.75**	0.343	0.85	**0.029**	**0.63**	**0.029**	**0.81**	**0.029**	**0.72**	0.114	0.87	0.057	0.88	0.886	0.95
Serine[a]	**0.029**	**0.59**	**0.029**	**0.56**	**0.029**	**0.39**	**0.029**	**0.66**	**0.029**	**0.58**	0.886	1.02	0.057	1.15	**0.029**	**0.68**
Threonine[a]	**0.029**	**0.75**	**0.029**	**0.71**	**0.029**	**0.49**	**0.029**	**0.74**	**0.029**	**0.71**	1.000	1.01	**0.029**	**1.34**	0.343	0.84
N,N-Dimethylglycine[a]	**0.029**	**0.69**	0.114	0.72	**0.029**	**0.53**	0.114	0.73	0.114	0.77	0.686	0.91	0.686	0.92	0.114	0.77
Glutamic acid[a]	**0.029**	**0.59**	**0.029**	**0.55**	**0.029**	**0.44**	**0.029**	**0.57**	**0.029**	**0.53**	**0.029**	**0.70**	**0.029**	**0.76**	**0.029**	**0.62**
Aspartic acid[a]	**0.029**	**0.44**	**0.029**	**0.38**	**0.029**	**0.30**	**0.029**	**0.47**	**0.029**	**0.40**	**0.029**	**0.69**	**0.029**	**0.78**	**0.029**	**0.45**
Alanine[a]	0.057	0.79	0.114	0.80	**0.029**	**0.59**	**0.029**	**0.81**	**0.029**	**0.72**	0.686	1.06	0.057	1.18	0.200	0.89
Glutamine[a]	0.114	0.67	0.114	0.68	**0.029**	**0.50**	0.343	0.78	**0.029**	**0.63**	0.886	0.94	0.486	1.09	0.343	0.76
4-Aminobutanoic acid[a]	0.114	0.79	0.114	0.81	0.057	0.71	**0.029**	**0.47**	**0.029**	**0.63**	**0.029**	**0.69**	0.057	0.72	**0.029**	**0.63**
N-acetyl-aspartic acid[a]	**0.029**	**0.40**	**0.029**	**0.31**	**0.029**	**0.32**	**0.029**	**0.40**	**0.029**	**0.43**	**0.029**	**0.58**	**0.029**	**0.48**	**0.029**	**0.26**
Asparagine[a]	**0.029**	**0.38**	**0.029**	**0.34**	**0.029**	**0.25**	**0.029**	**0.59**	0.343	0.58	0.686	0.87	0.114	0.77	**0.029**	**0.52**
Pyroglutamic acid[a]	**0.029**	**0.61**	**0.029**	**0.60**	**0.029**	**0.46**	**0.029**	**0.66**	**0.029**	**0.65**	0.200	0.90	0.486	0.92	**0.029**	**0.75**
Pyrrolidonecarboxylic acid[b]	**0.057**	**0.67**	0.057	0.67	**0.029**	**0.56**	**0.029**	**0.64**	0.200	0.73	0.486	1.12	0.200	1.18	0.200	0.87
Proline[a]	**0.029**	**0.62**	**0.029**	**0.63**	**0.029**	**0.45**	**0.029**	**0.66**	**0.029**	**0.61**	0.114	1.26	0.057	1.39	**0.029**	**0.67**
Ornithine[a]	**0.029**	**0.63**	**0.029**	**0.65**	**0.029**	**0.50**	**0.029**	**0.61**	0.057	0.72	0.057	0.86	0.886	1.03	**0.029**	**0.75**
Citrulline[a]	**0.029**	**0.55**	**0.029**	**0.58**	**0.029**	**0.45**	**0.029**	**0.70**	**0.029**	**0.58**	0.114	0.82	0.886	1.03	**0.029**	**0.66**
Urea[a]	0.200	0.89	0.686	0.91	**0.029**	**0.80**	**0.029**	**0.72**	**0.029**	**0.48**	**0.029**	**0.43**	**0.029**	**0.74**	**0.029**	**0.67**

**Table S1** Continued.																
Trans-4-hydroxyproline[a]	0.486	0.67	0.114	0.52	**0.029**	**0.45**	**0.029**	**0.56**	0.114	0.56	0.200	0.69	0.114	1.51	0.200	0.73
Valine[a]	**0.029**	**0.59**	**0.029**	**0.59**	**0.029**	**0.47**	**0.029**	**0.70**	**0.029**	**0.64**	0.057	0.86	0.343	1.08	**0.029**	**0.69**
Leucine[a]	**0.029**	**0.58**	**0.029**	**0.57**	**0.029**	**0.45**	**0.029**	**0.69**	0.057	0.69	0.486	0.94	0.486	1.11	**0.029**	**0.64**
Isoleucine[a]	**0.029**	**0.55**	**0.029**	**0.54**	**0.029**	**0.42**	**0.029**	**0.66**	**0.029**	**0.64**	0.200	0.88	0.114	1.06	**0.029**	**0.65**
Phenylalanine[a]	**0.029**	**0.62**	**0.029**	**0.58**	**0.029**	**0.46**	**0.029**	**0.65**	**0.029**	**0.59**	**0.029**	**0.80**	0.114	0.89	**0.029**	**0.63**
Tyrosine[a]	**0.029**	**0.35**	**0.029**	**0.36**	**0.029**	**0.38**	**0.029**	**0.55**	**0.029**	**0.49**	0.114	0.86	0.343	0.94	**0.029**	**0.46**
Dopa[a]	**0.029**	**7.53**	**0.029**	**7.44**	**0.029**	**2.79**	**0.029**	**9.50**	**0.029**	**7.47**	**0.029**	**5.03**	0.057	2.78	**0.029**	**8.03**
Tryptophan[a]	**0.029**	**0.29**	**0.029**	**0.27**	**0.029**	**0.20**	**0.029**	**0.40**	**0.029**	**0.36**	0.114	0.67	0.114	0.62	**0.029**	**0.31**
Methionine[a]	**0.029**	**0.62**	**0.029**	**0.57**	**0.029**	**0.44**	**0.029**	**0.63**	**0.029**	**0.56**	0.343	0.84	0.686	1.00	**0.029**	**0.58**
Lysine[a]	**0.029**	**0.51**	**0.029**	**0.57**	**0.029**	**0.40**	**0.029**	**0.62**	**0.029**	**0.59**	0.200	0.86	0.886	0.99	**0.029**	**0.64**
N-acetyl-lysine[b]	**0.029**	**0.50**	**0.057**	**0.52**	**0.029**	**0.38**	**0.029**	**0.61**	0.057	0.62	0.343	0.81	0.343	0.84	**0.029**	**0.63**
Histidine[b]	**0.029**	**0.31**	**0.029**	**0.35**	**0.029**	**0.27**	**0.029**	**0.44**	**0.029**	**0.36**	0.486	0.81	1.000	0.99	0.057	0.43
Mimosine[a]	**0.029**	**0.81**	**0.029**	**0.85**	**0.029**	**0.74**	**0.029**	**0.60**	0.686	0.96	0.886	0.97	**0.029**	**0.74**	**0.029**	**0.74**
Pantothenic acid[a]	**0.029**	**0.43**	**0.029**	**0.41**	**0.029**	**0.40**	**0.029**	**0.58**	**0.029**	**0.43**	**0.029**	**0.54**	**0.029**	**0.69**	**0.029**	**0.54**
Taurine[b]	**0.029**	**0.27**	**0.029**	**0.11**	**0.029**	**0.06**	**0.029**	**0.36**	**0.029**	**0.32**	0.114	0.64	0.114	0.58	**0.029**	**0.27**
Lipid metabolism																
Hexanoic acid[a]	1.000	0.94	0.057	0.66	**0.029**	**0.46**	0.343	0.79	0.200	1.18	0.343	1.13	0.686	1.07	0.886	1.05
Myristic acid[a]	**0.029**	**0.84**	0.057	0.88	**0.029**	**0.83**	0.114	0.87	0.057	0.81	0.057	0.88	0.114	0.90	**0.029**	**0.83**
Heptadecanoic acid[a]	0.486	0.95	0.200	0.91	0.114	0.87	0.057	0.90	**0.029**	**0.86**	0.057	0.88	0.114	0.89	0.886	0.98
Oleic acid[a]	0.200	0.93	0.343	1.05	0.343	0.94	0.114	0.87	1.000	1.00	0.686	0.98	**0.029**	**0.84**	0.686	1.03
Trans-9-octadecenoic acid[a]	0.343	0.91	1.000	1.02	0.343	0.94	0.114	0.90	0.343	0.92	0.114	0.89	**0.029**	**0.79**	0.343	0.94
Linoleic acid[a]	0.200	0.86	1.000	1.03	0.886	0.95	**0.029**	**0.73**	0.886	0.94	1.000	0.96	0.057	0.77	1.000	1.02
5,8,11,14,17-Eicosapentaenoic acid[a]	0.886	1.01	0.114	1.17	0.114	1.14	0.686	0.96	0.686	0.95	0.686	1.02	**0.029**	**0.83**	0.057	1.25
4,7,10,13,16,19-Docosahexaenoic acid[a]	0.686	1.04	**0.029**	**1.39**	0.057	1.17	1.000	1.01	0.200	0.93	0.200	0.92	**0.029**	**0.71**	0.057	1.11
Monosteari[a]	0.686	0.94	0.343	1.23	0.343	1.04	**0.029**	**0.74**	0.200	0.90	1.000	1.00	0.686	0.94	0.886	1.03

Table S1 Continued.

Ethanolamine[a]	0.686	0.95	**0.029**	**1.55**	0.486	1.14	0.343	0.90	0.343	1.17	0.200	1.19	**0.029**	**1.33**	0.200	1.43
O-phosphoethanolamine[a]	**0.029**	**0.55**	**0.029**	**0.50**	**0.029**	**0.43**	**0.029**	**0.48**	**0.029**	**0.53**	**0.029**	**0.60**	**0.029**	**0.56**	**0.029**	**0.40**
Glycerol[a]	**0.029**	**0.63**	**0.029**	**0.74**	**0.029**	**0.55**	**0.029**	**0.79**	**0.029**	**0.56**	**0.029**	**0.81**	**0.029**	**0.73**	**0.029**	**0.75**
Glycerol 3-phosphate[a]	**0.029**	**0.56**	**0.029**	**0.60**	**0.029**	**0.48**	**0.029**	**0.76**	**0.029**	**0.71**	**0.029**	**0.81**	0.114	0.82	**0.029**	**0.65**
Cholesterol[a]	0.343	0.91	0.886	0.95	0.486	0.90	0.486	0.84	0.686	0.95	0.343	0.93	**0.029**	**0.72**	0.886	0.96
Dihydrocholesterol[b]	**0.029**	**0.81**	0.057	0.89	0.057	0.84	0.057	0.77	0.114	0.91	0.057	0.88	**0.029**	**0.67**	0.486	0.93
Nucleoside metabolism																
Hypoxanthine[a]	**0.029**	**0.31**	**0.029**	**0.28**	**0.029**	**0.26**	**0.029**	**0.26**	**0.029**	**0.34**	0.057	0.65	0.057	0.59	**0.029**	**0.40**
Uric acid[a]	**0.029**	**0.40**	**0.029**	**0.31**	**0.029**	**0.32**	**0.029**	**0.21**	**0.029**	**0.31**	**0.029**	**0.38**	**0.029**	**0.41**	**0.029**	**0.21**
Inosine[a]	**0.029**	**0.17**	**0.029**	**0.24**	**0.029**	**0.24**	**0.029**	**0.37**	**0.029**	**0.25**	**0.029**	**0.09**	**0.029**	**0.12**	**0.029**	**0.15**
Adenine[a]	**0.029**	**0.66**	**0.029**	**0.58**	**0.029**	**0.56**	**0.029**	**0.52**	**0.029**	**0.66**	0.486	0.96	**0.029**	**0.84**	**0.029**	**0.35**
Others																
Alpha-Tocopherola[a]	0.486	0.89	0.886	1.01	1.000	1.01	0.057	0.81	0.486	0.91	**0.029**	**0.77**	**0.029**	**0.61**	0.114	0.78
Monomethylphosphoric acid[a]	0.057	0.78	0.057	0.79	**0.029**	**0.70**	0.057	0.78	0.343	0.91	0.686	0.95	0.114	0.75	0.114	0.75
Hydroxylamine[b]	0.686	1.13	0.057	1.44	**0.029**	**1.43**	0.200	1.30	0.686	0.97	0.686	0.88	1.000	1.01	1.000	1.00
Phospate[a]	**0.029**	**0.76**	**0.029**	**0.76**	**0.029**	**0.68**	**0.029**	**0.78**	**0.029**	**0.83**	**0.029**	**0.81**	**0.029**	**0.77**	**0.029**	**0.58**

[a] Identified by library search and further verified by standard references according to the mass spectral, retention time and retention index. [b] Identified by library search according to the mass spectral. [c] C, the control group. The two-tailed Mann-Whitney U test was performed to evaluate the significant level between the control and treatment groups. Bold red fonts: metabolites were significantly increased in the treatment group compared to the control group; Bold red fonts: metabolites were significantly decreased in the treatment group compared to the control group. Raw peak table of the metabolites were provided in Table S2.
